# RNMT-dependent RNA cap methylation in health and disease

**DOI:** 10.1042/BCJ20253170

**Published:** 2025-09-09

**Authors:** Jacquie G. Mills, Lydia A. Hepburn, Victoria H. Cowling

**Affiliations:** 1 Cancer Research UK Scotland Institute, Glasgow, G61 1BD, U.K.; 2 School of Cancer Sciences, University of Glasgow, Glasgow, G61 1QH, U.K

**Keywords:** gene regulation, RNA, RNA methylation, RNA processing, transcription, translation

## Abstract

RNA cap formation on RNA polymerase II transcripts is regulated by cellular signalling pathways during development and differentiation, adaptive and innate immune responses, during the cell cycle and in response to oncogene deregulation. Here, we discuss how the RNA cap methyltransferase, RNA guanine-7 methyltransferase (RNMT), functions to complete the 7-methyl-guanosine or ^m7^G cap. The mechanisms by which RNMT is regulated by signalling pathways, co-factors and other enzymes are explored. The ^m7^G cap protects RNA pol II-transcribed RNA from the initiation of transcription and recruits proteins that mediate RNA processing including splicing, 3′ cleavage and polyadenylation, nuclear export and translation initiation. Regulation of RNMT has gene-specific impacts with implications for cell function, cell physiology and cell fate decisions.

## Introduction

**Figure 1:**
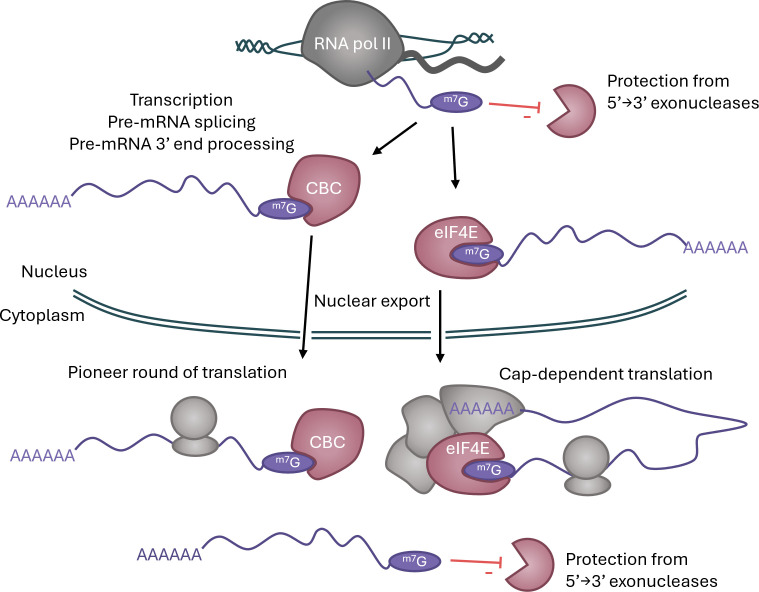
Functions of the ^m7^G cap The ^m7^G cap is added early in transcription to RNA pol II transcripts, preventing degradation of the nascent transcript in the nucleus. Recruitment of CBC by the ^m7^G cap promotes transcriptional processing including splicing and polyadenylation, followed by nuclear export. The ^m7^G cap is bound by eIF4E and other complexes for nuclear export. In the cytoplasm, the ^m7^G cap continues to stabilise the transcript. CBC binding is required for the pioneer round of translation, with subsequent translation mediated by eIF4E. (CBC, cap binding complex; eIF4E, eukaryotic translation initiation factor 4E; RNA pol II, RNA polymerase II.)

Messenger RNA (mRNA) and other RNA polymerase (pol) II-transcribed RNAs are marked by the addition of a RNA cap, which controls recruitment of specific proteins to the 5′ end. The ^m7^G cap, which is part of the RNA cap found in all eukaryotes, protects RNA pol II-transcribed RNA from interaction with nucleases during transcription and throughout the lifetime of the RNA. The ^m7^G cap also recruits proteins that mediate RNA processing including splicing, 3′ cleavage and polyadenylation, nuclear export and translation initiation (Figure 1) [1–3]. Thus, the ^m7^G cap is required for the maturation and functions of mRNA. Formation of the ^m7^G cap is a dynamic process that is regulated by cellular signalling pathways during development and differentiation, adaptive and innate immune responses, during the cell cycle and in response to oncogene deregulation. The impact of regulation of RNA cap methylation is gene- and cell lineage-specific, with implications for cell function, cell physiology and cell fate decisions.

## RNA cap formation: structure and capping reactions

### ^m7^G cap formation

**Figure 2:**
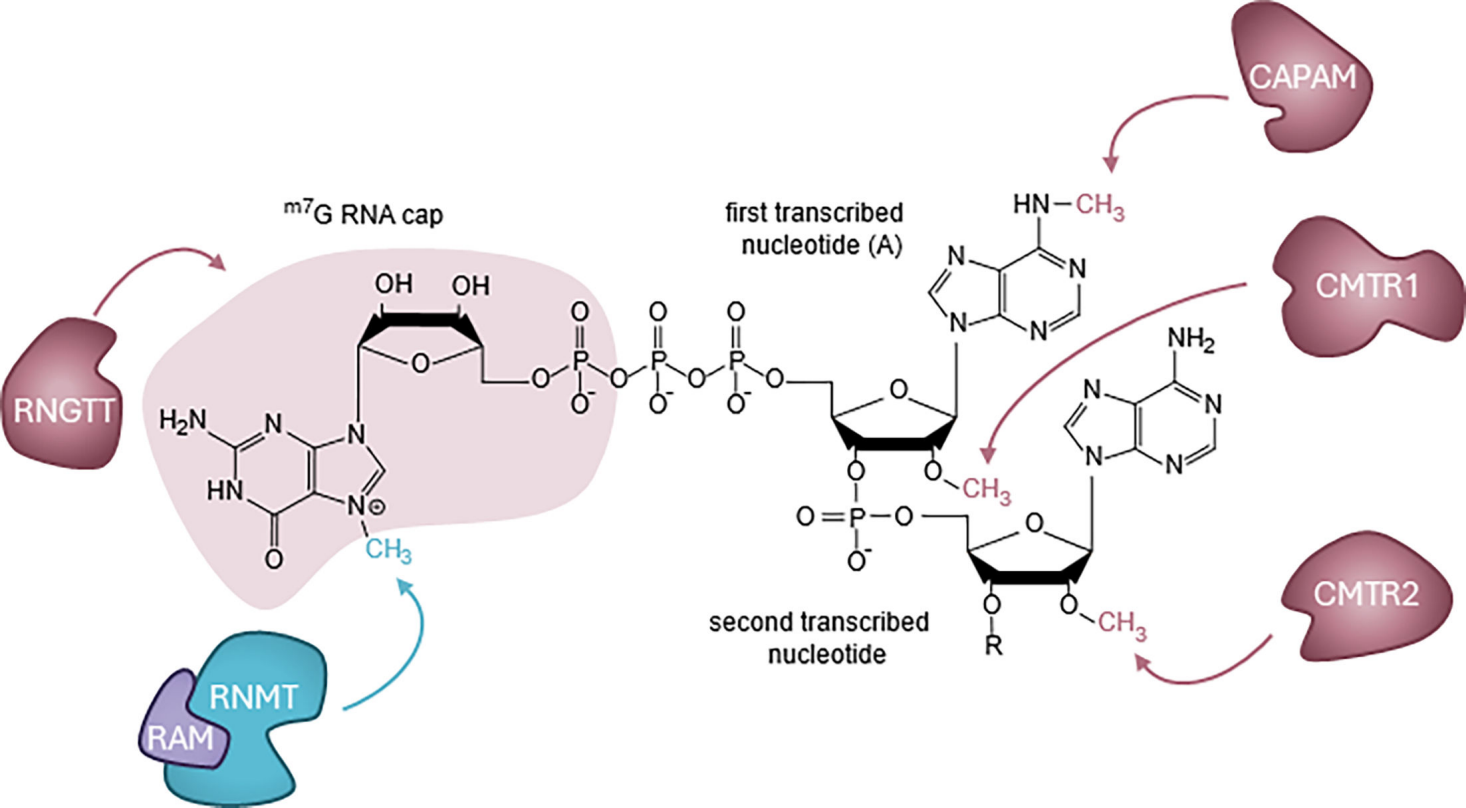
The RNA cap and capping enzymes The RNA cap structure consists of an inverted guanosine connected by a 5′-5′ triphosphate bridge to the first transcribed nucleotide. RNA cap methylations are catalysed by specific RNA cap methyltransferases. Cap guanosine-7 methylation by RNMT, O2 methylation of the first transcribed nucleotide ribose by CMTR1, O2 methylation of the second transcribed ribose nucleotide by CMTR2. If the first transcribed nucleotide is an adenosine, ^m6^A methylation by CAPAM may occur. RNMT is depicted with its activating subunit RAM (RNMT-activating miniprotein). CAPAM, cap adenosine N6-methyltransferase; CMTR1, cap methyltransferase 1; CMTR2, cap methyltransferase 2; RNMT, RNA cap guanine-7 methyltransferase; RAM, RNMT-activating miniprotein.

**Figure 3:**
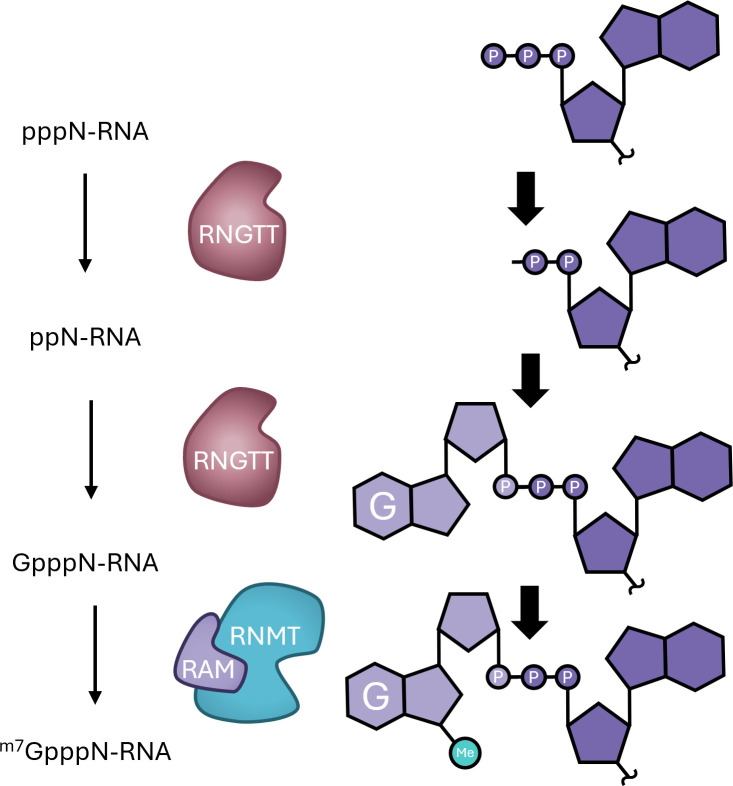
^m7^G cap formation RNGTT triphosphatase activity removes the terminal phosphate of the first transcribed nucleotide and guanylyltransferase activity adds GMP to form the inverted guanosine cap (GpppN, N is the first transcribed nucleotide). RNMT catalyses N7 methylation of the guanosine cap to form the ^m7^G cap (^m7^GpppN). RNMT is depicted with its activating subunit, RAM. (RNGTT, RNA guanylyltransferase and 5′-phosphatase; RNMT, RNA cap guanine-7 methyltransferase; RAM, RNMT-activating miniprotein)

The ^m7^G cap consists of N7-methyl-guanosine linked via an inverted 5′-5′ triphosphate bridge to the first transcribed nucleotide, a structure also known as cap0 and denoted ^m7^GpppN (N is the first nucleotide, Figure 2) [4]. The ^m7^G cap is formed by three sequential reactions on nascent RNA as it emerges from the RNA pol II complex (Figure 3) [2,3]. Nascent RNA is synthesised with a triphosphate at the 5′ end (pppN-RNA). The RNA triphosphatase hydrolyses the γ-phosphate of the first transcribed nucleotide, producing ppN-RNA. The RNA guanylyltransferase hydrolyses GTP, producing GMP which is then transferred to the 5′ diphosphate of nascent RNA to create guanosine-capped RNA, GpppN-RNA. The guanine-7 methyltransferase methylates the N7-amine of the guanosine cap to create cap0, ^m7^GpppN-RNA. The first transcribed nucleotide can also be methylated on the ribose at the O2 position to form cap1 (^m7^GpppN_m_-RNA), the major cap structure found in metazoan cells [5,6]. In mammals and some other eukaryotes, the second transcribed nucleotide can be methylated at the ribose O2 position. Methylation of the N7 on the cap guanosine and O2 ribose on the first and second transcribed nucleotides results in formation of cap2, ^m7^GpppN_m_N_m_-RNA. Both O2 methylation positions are utilised for self-recognition as part of the innate immune response [5,6]. O2 methylation of the first transcribed nucleotide is associated with transcription, splicing and translation, whereas O2 methylation of the second transcribed nucleotide is linked to protein biosynthesis and mRNA ageing [7–12]. If the first transcribed nucleotide is adenosine, this can be methylated on the N6 position, to produce the cap structure ^m7^Gppp^m6^A_m_ [13–16]. In addition, non-canonical metabolite caps are present at low but significant levels in mammalian cells [15,17].

In addition to mRNA, other RNA pol II-transcribed RNAs, such as pre-microRNA (miRNA), small nuclear RNA (snRNA) and long non-coding RNA (lncRNA), receive the ^m7^G cap. These RNAs are processed differently depending on the type of RNA. Pre-miRNA is cleaved during the maturation process which removes the ^m7^G cap [18]. In snRNA, the ^m7^G cap is further methylated to create the 2,2,7-trimethylguanosine cap [19,20]. LncRNAs are diverse and can undergo differential processing in response to cellular signals, with a subset retaining ^m7^G cap-like structures [21]. Here, we focus on the regulation and function of the ^m7^G cap on mRNA in mammalian cells.

### The RNA capping enzymes

The mechanism of ^m7^G cap addition is conserved across eukaryotes, although the configuration of the RNA capping enzymes differs between species [22,23]. In metazoans, the triphosphatase and guanylyltransferase activities are contained on one polypeptide in the bifunctional enzyme RNA guanylyltransferase and 5′-phosphatase (RNGTT). RNGTT binds to the RNA pol II complex, positioned to interact with the 5′ end of the RNA as it emerges from the complex [24]. The cap guanosine N7-methyltransferase, RNA guanine-7 methyltransferase (RNMT), is encoded on a separate enzyme [25–27]. RNMT has a co-activator, RNMT-activating miniprotein (RAM, FAM103A1, RAMAC) [28,29]. Yeast species have RNA cap guanosine N7 methyltransferases homologous to RNMT but a RAM homologue has not been identified [23,28].

Methylation of the first and second transcribed nucleotides (also described as cap-adjacent nucleotides) is catalysed by specific RNA cap methyltransferases (Figure 2). O2 methylation of the first and second transcribed nucleotides on the ribose is catalysed by cap methyltransferase 1 (CMTR1) [30,31] and cap methyltransferase 2 (CMTR2) [32], respectively. CMTR1 and CMTR2 are essential for mouse embryonic development [33]. Cap m^6^A methylation is catalysed by cap adenosine N6-methyltransferase (CAPAM, previously PCIF1) and is involved in transcript stability, translation and other RNA processing events [13,14,34–36]. CAPAM, CMTR1 and, as described later, RNMT, have functions in transcription, independent of their methyltransferase activity [11,37,38]. Evolution of these additional functions of the RNA cap methyltransferases may have been facilitated by capping occurring co-transcriptionally.

As the RNA cap methyltransferases all bind to and methylate cap dinucleotides (GpppN-RNA), addition of the guanosine cap must occur prior to cap methylation. However, the order of individual cap methylations is not defined and may be different for specific RNAs or under certain scenarios. A series of RNA cap structures are found on RNA pol II transcripts. Cellular mRNA enriched via the (A) tail have high levels of ^m7^G and first transcribed nucleotide N_m_ cap modifications, but individual RNAs can have variable amounts of these modifications [15,16,39]. When RNA caps on total cellular RNAs are analysed by mass spectrometry, more variation in the cap structures is observed, although ^m7^G caps predominate [40]. This indicates that cap guanosine methylation occurs early during mRNA processing prior to poly(A) tail addition and that RNA caps are methylated in variable order. Furthermore, since mRNA without ^m7^GpppN_m_ is detected at low levels in mRNA, it is likely to be unstable especially prior to poly(A) tail addition.

## ^m7^G cap function: controlling interaction with cap-binding proteins

Since RNGTT is recruited directly to the RNA pol II complex, the ^m7^G RNA cap is only found on RNA pol II-transcribed RNA [24]. The methylation status of the RNA cap nucleotides controls which cap-binding proteins are recruited to it and which are inhibited from binding [2,3]. The initial function of the ^m7^G cap is to protect the transcript from degradation during transcription. Unmethylated caps on nascent transcripts are removed by decapping exoribonucleases [41,42], and other decapping enzymes also recognise and subsequently remove hypomethylated caps [43]. The ^m7^G cap further protects mature RNA pol II transcripts from cytoplasmic 5’-3’ exonucleases [44]. There are multiple mechanisms controlling degradation of cytoplasmic mRNA at the end of its lifetime [45]. mRNA degradation is often initiated by deadenylation followed by decapping. The most well-characterised decapping enzyme for this activity is the NUDIX hydrolase decapping mRNA 2 (DCP2), although other NUDIX hydrolase family members also possess specificity towards the ^m7^G cap [43]. Similar to the regulation of RNA capping, cellular signalling pathways can affect the rate and specificity of decapping. Decapping and its regulation are reviewed elsewhere [43,45–47].

Most functions of the ^m7^G cap in mRNA processing and translation are mediated by cap-binding proteins. Here, we discuss the most well-characterised cap-binding proteins and their role in the mRNA life cycle.

### Cap-binding complex (CBC)

CBC mediates many of the nuclear functions of the ^m7^G cap [29,48]. CBC is a heterodimer of nuclear cap-binding protein 1 (NCBP1) and nuclear cap-binding protein 2 (NCPB2) [49–53]. NCBP2 contains the cap-binding pocket and NCBP1 increases NCBP2 affinity for the cap [54,55]. CBC has a preference for the ^m7^G cap over the unmethylated G cap [56]. CBC associates with the ^m7^G cap shortly after its formation to promote transcription elongation [57], splicing [58], 3′ cleavage [59] and nuclear export [51]. In the cytoplasm, CBC is required for the pioneer round of translation prior to translation initiated by the translation initiation factor eIF4E [60].

### Eukaryotic initiation factor 4F (eIF4F)

Eukaryotic translation predominantly initiates with ribosome recruitment to the 5′ end of mRNA [61]. This is facilitated by the translation initiation factor eIF4F, a heterotrimer of the cap-binding protein eIF4E, the scaffold protein eIF4G and the DEAD-box RNA helicase eIF4A1 [62]. eIF4E has a preference for the ^m7^G cap over the unmethylated G cap [63]. Multiple cell signalling pathways influence the function of the eIF4F complex including mechanistic/mammalian target of rapamycin complex 1 (mTORC1) and mitogen-activated protein kinase (MAPK) pathways [61,64]. mTORC1 co-ordinates protein synthesis with environmental stimuli by phosphorylating downstream effectors including eIF4E-binding proteins (4E-BPs). Unphosphorylated 4E-BPs compete with eIF4G for eIF4E binding, repressing translation initiation when mTORC1 is inactivated [65]. When the MAPK pathway is activated by mitogen signalling, MAPKs phosphorylate and activate MAPK-interacting kinases, which phosphorylate eIF4E at serine-209, thus permitting and promoting translation [66]. The eIF4E paralogues 4EHP and eIF4E3 also interact with the cap to regulate translation under cellular conditions such as stress [67]. As discussed later, eIF4E also promotes processing and nuclear export of specific RNAs [68].

### La-related protein 1 (LARP1)

Protein synthesis pathways are coupled to nutrient and energy availability by the mTORC1 pathway. Ribosomal proteins and several translation initiation factors share a 5′ terminal oligopyrimidine (5′ TOP) motif, characterised as a cytosine in the +1 position followed by a run of pyrimidines [69]. TOP mRNAs are regulated by the specialised cap-binding protein LARP1 [69,70]. LARP1 contains multiple RNA-binding domains, of which the DM15 domain directly recognises the ^m7^G cap and the adjacent 5′ TOP motif [71–78]. LARP1 recognition of the RNA cap is dependent on cap guanosine-7 methylation [79]. LARP1 also interacts with the 3′ end of transcripts via direct interactions with the poly(A) sequence and poly(A)-binding protein [72,77,80–83]. LARP1 may simultaneously bind both the 5′ TOP motif and the poly(A) tail via the La module, resulting in mRNA circularisation [83].

LARP1 is regulated by mTORC1 to influence TOP mRNA stability and translation [71,74,77,78,81,84,85]. The role of LARP1 in TOP mRNA stability and translation regulation is the subject of debate and its precise function may differ depending on the transcript, cell lineage and status [70,86,87]. Under energy-restricted conditions, TOP mRNAs are translationally repressed. Since the LARP1 DM15 domain has a higher affinity for the ^m7^G cap of TOP mRNAs than eIF4E, it outcompetes eIF4E for cap binding, leading to translational repression [74,76]. The elongation initiation factor eIF4A1 has been identified as an essential partner for LARP1 in translation repression, revealing further interplay between LARP1 and the canonical eIF4F cap-binding complex [88]. Phosphorylation of LARP1 by mTORC1 inhibits LARP1 binding to TOP mRNAs [71,77,78,89]. Simultaneously, mTORC1 phosphorylates 4E-BP, leading to release of eIF4E from this repressive complex, to further drive TOP RNA translation [[[90,91]]].

In spite of its repressive role in TOP mRNA translation, LARP1 can promote cell growth/survival [78,80,84,90,92–96]. LARP1 stabilises TOP mRNAs [72,73,77,90,92]. Binding of LARP1 to the cap may prevent decapping of TOP mRNAs leading to transcript stabilisation [45,86]. By allowing the cell to maintain a pool of stabilised, but translationally repressed TOP mRNA during periods of nutrient deprivation or developmental inhibition of cell growth, LARP1 supports rapid translation recovery upon mTORC1 activation [71,78,84].

There are many other cap-binding proteins that have roles in mRNA synthesis, processing, localisation, function and degradation. They are extensively discussed elsewhere [2,3,97].

## RNMT, the ^m7^G cap methyltransferase

**Figure 4:**
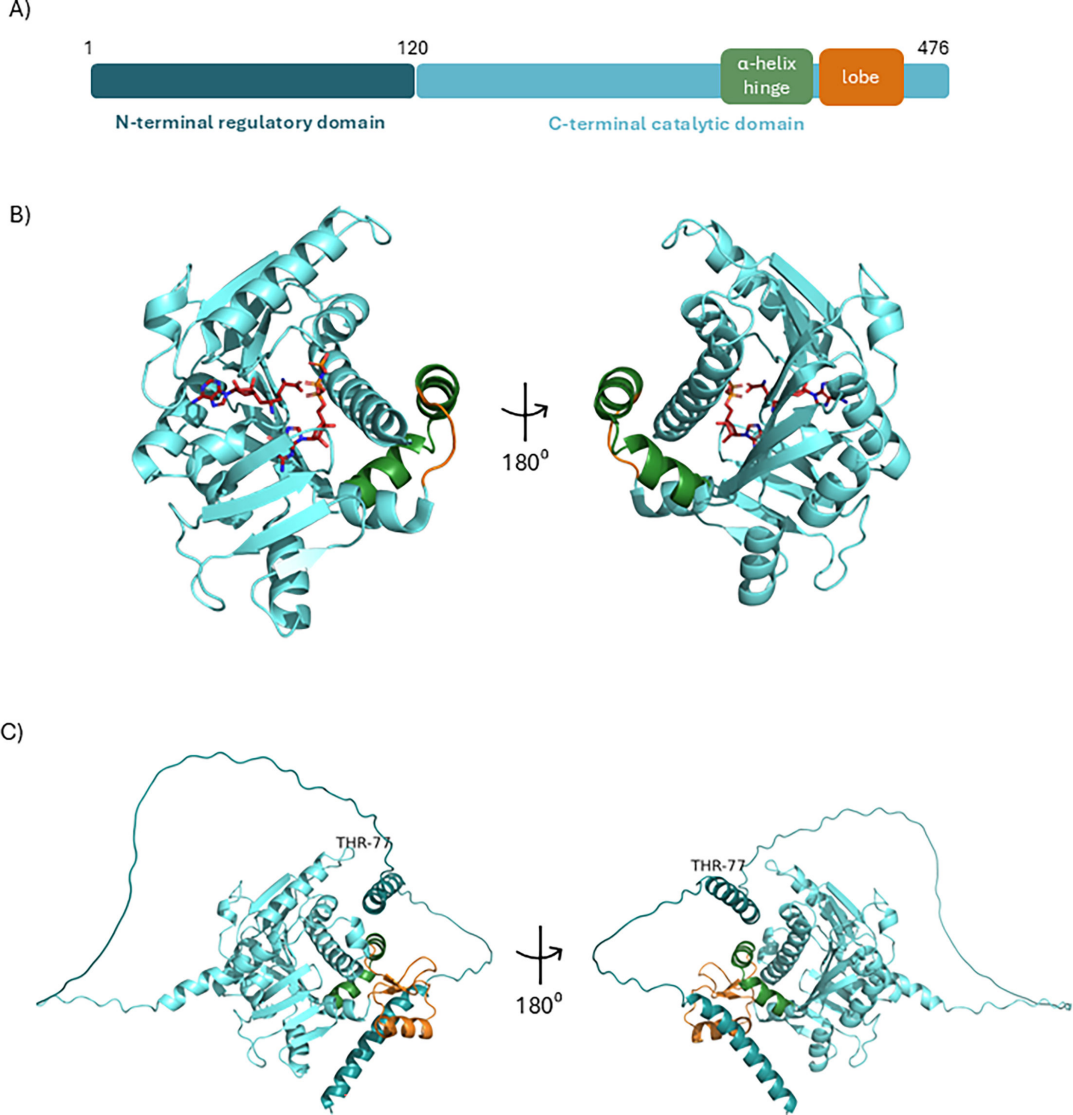
RNMT domains and structure (A) RNMT has an N-terminal regulatory domain and a C-terminal catalytic domain. The N-terminal domain is the site of post-translational modification and is required for nuclear localisation, importin-α interaction and chromatin recruitment. The C-terminal catalytic domain is the site of methyltransferase activity, RNA binding and RAM binding. (**B**) Crystal structure of RNMT 170–476. Light cyan is the methyltransferase domain, green the α-helix hinge, orange the RNMT lobe. PDB ID: 8Q9W. (**C**) AlphaFold predicted structure of RNMT. Light cyan is the methyltransferase domain, green the α-helix hinge, orange the RNMT lobe and dark cyan is the N-terminal domain.

RNMT is the predominant, if not the only, ^m7^G cap methyltransferase in mammals [26,98,99]. On experimental reduction in RNMT expression, there is a corresponding reduction in ^m7^G cap methyltransferase activity in cell extracts [79,100–102]. Another ^m7^G cap methyltransferase has not been isolated from cell extracts or identified by computational analysis, indicating that RNMT is the only mammalian enzyme with this function. Human RNMT has a methyltransferase domain (residues 121–476) and a non-catalytic N-terminal domain (residues 1–120) (Figure 4A) [103]. RNMT catalyses the transfer of a methyl group from the methyl donor, *S*-adenosyl methionine (SAM), to the N7 position of the unmethylated guanosine cap (Figure 2). The structure of RNMT has been investigated using x-ray crystallography, molecular dynamics simulations and small molecule inhibition (Figure 4B) [104–107]. Human RNMT has a well-conserved canonical Class I methyltransferase fold of alternating β-strands (β1-β7) and α-helices (α-helix A-E) [104,105,108]. Human RNMT also contains a flexible lobe structure (residues 416–456), which is unique to vertebrates and absent from lower eukaryotes [104,105].

The RNMT SAM-binding pocket consists of strands β1-β2, and the cap-binding pocket is formed from strands β8-β9 [104,105]. Within these pockets, multiple stabilising interactions between the substrates and enzyme form, including co-ordination of both substrates by RNMT residues that interact with both SAM and the cap simultaneously. The substrate-binding pockets have high binding affinity for their substrates, unmethylated cap and SAM, with lower binding affinity for the products, methylated cap (^m7^GpppN) and *S*-adenosyl homocysteine (SAH) [107,108]. Kinetic characterisation indicates that RNMT binds SAM before binding to the unmethylated cap [107], although molecular dynamics modelling indicates that cap binding can enhance SAM recruitment [104,105].

RNMT positions the cap and SAM for optimal methyl transfer [28,104,105,107,108]. The RNMT lobe (residues 416–456), α-helix hinge (α-helices G and H, residues 395–415) and α-helix A are important for substrate binding [104]. The RNMT lobe is a highly flexible and dynamic structure that likely co-evolved with RAM in vertebrates to regulate methyltransferase activity. The adjacent surfaces of the lobe and hinge are positively charged, resulting in electrostatic repulsion and destabilisation of both the lobe and hinge. As the hinge directly contacts both the lobe and helix A of the active site, increased mobility of the hinge also increases the mobility of helix A, allowing it to adopt multiple conformations incompatible with substrate binding. Thus, flexibility of the RNMT lobe allosterically destabilises active site components to decrease binding of RNMT to its substrates.

The non-catalytic N-terminal domain is divergent between RNMT homologues, potentially allowing for differential functions and roles of RNMT to evolve [103]. This highly disordered region has not been structurally characterised, but modelling with AlphaFold indicates that it has the potential to form an α-helix that interacts with the methyltransferase domain (Figure 4C) [109,110]. Nuclear localisation can be mediated by lysine-based nuclear localisation sequences present at positions 80, 103 and 126, spanning the methyltransferase and N-terminal domains independently [100,111]. RNMT nuclear import is dependent on importin-α [112]. RNMT binding to RNA is mediated by residues 144–200 in the methyltransferase domain [113]. The RNMT N-terminal domain also negatively regulates methyltransferase activity which can be neutralised by phosphorylation [111,112,114]. Conformational plasticity of the disordered regions of RNMT may facilitate interactions with RNAs and proteins which mediate catalytic-independent functions (discussed later).

### RAM, the RNMT co-activator

**Figure 5:**
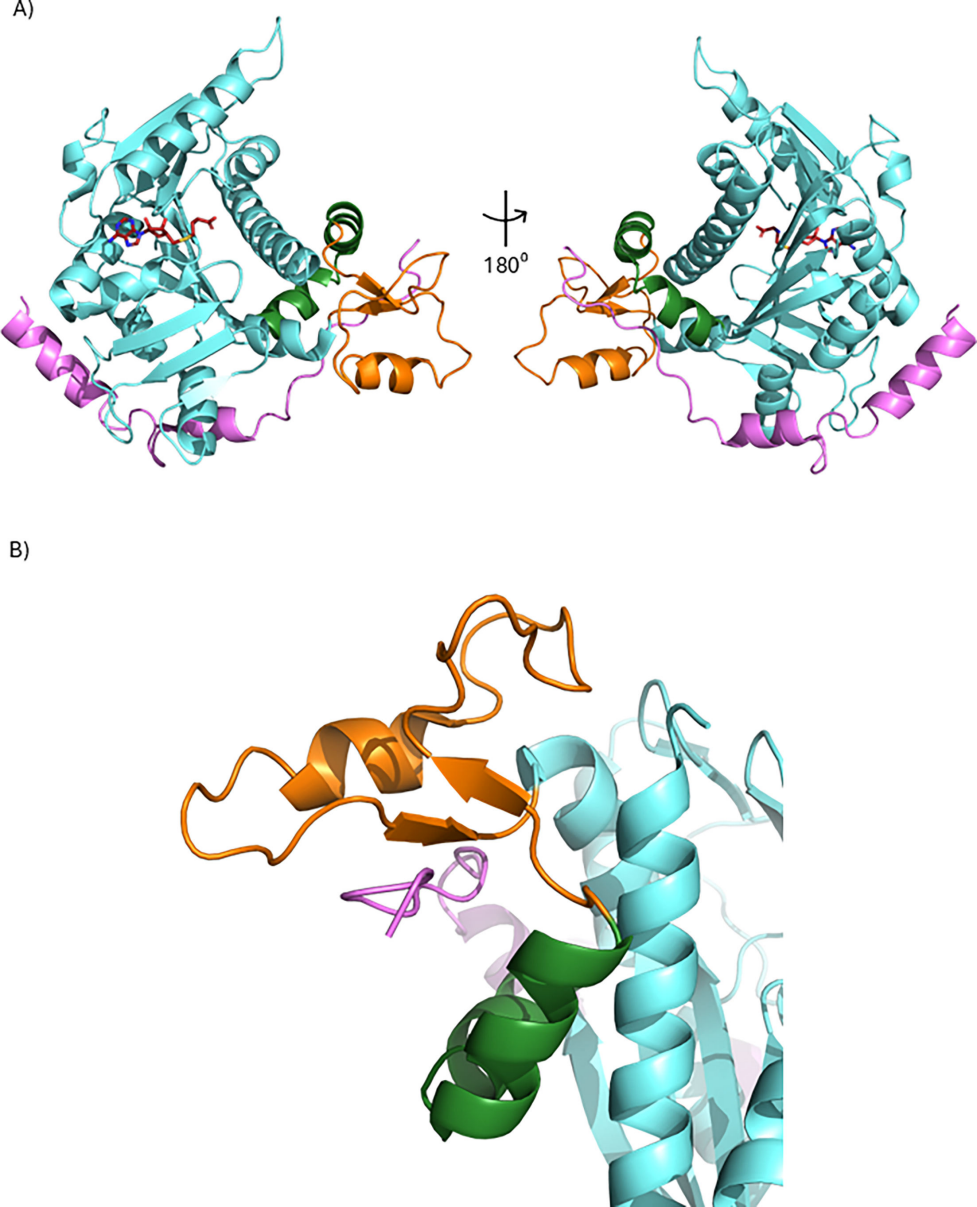
Structure of RNMT and RAM (A) Crystal structure of RNMT 167–476 with RAM 3–44. PDB ID: 5E8J. Light cyan is the methyltransferase domain, green the α-helix hinge, orange the RNMT lobe, purple is RAM. **B**) Interaction of the RNMT lobe, α-helix hinge, RAM and helix A.

RNMT is present in a complex with its co-activator RAM ( Figure 5A) [28]. RAM was identified as a 118 amino acid, uncharacterised protein in RNMT complexes purified from HeLa cells and HEK293T cells. In these cells and other cancer cell lines in which they have been investigated, RNMT and RAM are found in complex with each other with minimal detection of their monomeric forms. RNMT and RAM are co-translated and stabilise each other; monomeric RNMT and RAM are degraded by the proteasome [28,29]. Accordingly, RNMT and RAM expression levels are linked in many cell lines whereby experimentally altering expression of one regulates the stability of the other [28,79,102,115]. However, RNMT and RAM stability and functions can be partially uncoupled as judged by protein, RNA and phenotypic analysis following experimental reduction in gene expression in embryonic stem cells (ESCs) and T cells [79,115,116]. This indicates alternative proteins or post-translational modifications may stabilise RNMT in the absence of RAM in these cells.

RAM is a predominantly nuclear protein containing a conserved RNMT-activation domain (RAD, residues 1–55), NR-rich RNA-binding (residues 56–90) and a QYP-rich nuclear localising region (NLS, nuclear localisation signal, residues 91–118) [28,29]. RAM increases RNMT methyltransferase activity *in vitro* and in cells [28]. Direct interaction of the RNMT methyltransferase domain (121-476) and the RAM RAD promotes methyltransferase activity and protects RNMT from proteasomal degradation [28,29,104,105]. RNA binds to the NR-rich region *in vitro* [28,38]; however, all domains of RAM are required for interaction with RNA in cells [38]. In the complex intracellular environment, the additional domains of RAM may be required to support the subcellular localisation or protein:protein interactions, which support RNA binding. The C-terminal QYP-rich region is required for nuclear localisation [29]. The QYP-rich region contains two PY NLS sequences, PY1 (P^98^Y) and PY2 (P^114^Y), that are recognised by Kapβ2 for nuclear entry. Although RNMT and RAM stabilise each other, including during translation, and are present in cells as a complex, it is notable that they both have nuclear localisation domains, which may either ensure robust nuclear localisation of the complex or support nuclear localisation of the monomers.

### RAM impact on RNMT activity

RAM binding increases RNMT methyltransferase activity [28,104,105]. The RAM RAD contains two α-helices (residues 4–14 and 24–31), which bind to RNMT by an electrostatically driven interaction [104]. RAM binds distal to the active site. The RAM negative patch (residues 35–45) binds to the positively charged surface groove formed by the lobe and hinge of RNMT, neutralising repulsive electrostatic interactions between these regions (Figure 5B). This neutralisation of like-charge repulsion stabilises helix A and other active site components, permitting substrate binding. Hence, RAM enhances RNMT catalysis by stabilising conformations favourable for substrate binding and subsequent catalysis.

### RNMT-RAM recruitment to capped RNA

RNMT is inefficiently recruited to unmethylated, guanosine-capped RNA in the absence of RAM [28,38,107,113]. Recombinant RNMT monomer does not bind efficiently to RNA [113], whereas RAM monomer interaction with RNA can be detected *in vitro* [28,29]. The RNMT-RAM complex has enhanced RNA binding compared with either RNMT or RAM alone. In yeast, a RAM protein is not required for RNA cap methylation, although other activators of yeast ^m7^G cap methyltransferases may be present [28,117]. The RAM-RNA-binding domain is not required for RAM to stimulate RNMT methyltransferase activity; however, it may increase the recruitment to specific RNA in cells and thus enhance their methylation [28,38].

Despite the inefficient recruitment of RNMT to RNA, RNMT targets specific transcripts for cap methylation and stabilisation [38,101,118–122]. Weak interaction of RNMT and RNA may be optimal for maintaining RNA cap methylation of the transcriptome. Recruitment to specific transcripts can occur indirectly (see later) as well as via specific RNA elements with enhanced affinity for RNMT. Certain transcripts, such as cyclin D1, may be directly recognised by a cap sensitivity element (CapSE) in the 3′ UTR (untranslated region) [122].

### RNMT-RAM recruitment to chromatin

Multiple mechanisms of RNMT recruitment to the RNA cap are possible. The N-terminal domain enhances recruitment to chromatin where it interacts with a number of chromatin and transcription-associated proteins and complexes [111,123]. However, the RNMT–chromatin interaction is weak and only robustly detected upon high levels of RNMT and RAM expression [11,111,123]. Upon the expression of tagged RNMT and CMTR1 from the endogenous locus, recruitment of CMTR1 to the transcription start site was detected at over 700 genes, whereas RNMT was only detected above threshold at 20 genes [11]. RNMT may have transient interactions with chromatin-associated proteins below the threshold of detection.

Although the capping enzymes RNGTT, CMTR1 and CAPAM bind to the phosphorylated RNA pol II C-terminal domain (CTD), a direct interaction of RNMT with RNA pol II has not been reported [13,24,25,30,40,99,124–126]. An interaction between RNMT and RNGTT has been observed under certain conditions, which may aid recruitment of RNMT to RNA pol II [99,127]. RNMT also interacts with RNA pol II elongation factors and chromatin associated factors such as the PAF (Polymeras-associated Factor) complex [38]. In lieu of an interaction between RNMT and RNA pol II, RNMT:protein interactions or RNMT:RNA interactions may facilitate RNMT recruitment to the guanosine cap during the initial stages of transcription. Alternatively, since RNMT-RAM is highly abundant and active, diffusion of RNMT may be sufficient for cap guanosine-7 methylation without the need for a docking interaction for many transcripts [24,111,126].

### RAM stabilisation of RNMT

RAM protein stabilises RNMT protein [28,29]. Knocking down RAM reduces RNMT protein levels, but not RNMT transcript levels. Conversely, knocking down RNMT results in reduced RAM protein. As discussed, the interaction between RNMT and RAM protects both subunits from proteasomal degradation [28,29]. Simultaneous up-regulation of RNMT and RAM occurs during T cell activation; these proteins are required to stabilise each other in T cells [79,115].

The co-dependency of RNMT and RAM expression is partially uncoupled in ESCs [116]. RNMT-RAM expression is high in ESCs where it is required for the expression of pluripotency-associated gene transcripts and other transcripts [116,128]. During differentiation, up-regulation of ERK1/2 activity results in phosphorylation of RAM at serine-36 (Ser36), targeting it for ubiquitination and proteasome-mediated degradation. RNMT is also partially repressed by this mechanism but not entirely destabilised, suggesting the presence of another mechanism of RNMT protein stabilisation in ESCs such as an alternative stabilising co-factor or post-translational modification. Furthermore, in certain organs, RNMT is well-expressed while levels of RAM are relatively low [116].

### Non-catalytic function of RNMT-RAM

**Figure 6:**
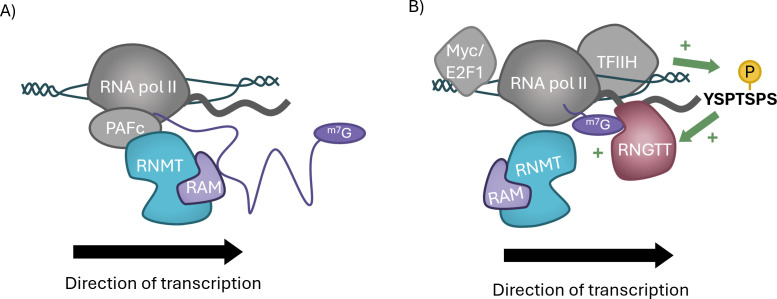
RNMT-RAM are involved in transcription by RNA pol II (A) RNMT-RAM supports transcription elongation by interactions with RNA pol II elongation factors such as the PAF complex. (**B**) During transcription initiation, ^m7^G cap formation by RNMT-RAM is promoted by the transcription factors Myc and E2F1. (E2F1, E2F transcription factor 1; Myc, Myc proto-oncogene, bHLH transcription factor; PAFc, polymerase-associated factor 1 complex; RNA pol II, RNA polymerase II; RNMT, RNA guanine-7; RAM, RNMT activating miniprotein; TFIIH, transcription factor II H)

RNA cap formation and transcription elongation are coupled in mechanisms described as a checkpoint model, whereby transcription elongation can only occur upon successful cap formation [129]. Contributing to this checkpoint mechanism is the requirement of RNMT-RAM in transcription elongation, including functions independent of its catalytic activity [38]. Methyltransferase-dead RNMT partially rescues mRNA synthesis in HeLa cells depleted of RNMT, and RNMT-RAM enhances transcription in isolated nuclei. RNMT-RAM binds across the full length of pre-mRNA and interacts with transcription-associated factors such as the PAF complex. In cells, inhibition of RNMT-RAM results in decreases in RNA pol II and PAF complex occupancy across the gene. Furthermore, Repression of RNMT-RAM reduces transcription in correlation with level of RNMT-RAM-RNA binding. RNMT-RAM promotes recruitment of elongation factors to RNA pol II, facilitating the transition from transcription initiation to elongation (Figure 6A).

### Regulation of RAM

Regulation of RAM is a mechanism by which cellular signalling pathways influence RNMT expression, function and activity [28,79,115,116]. RAM expression is up-regulated following T cell activation which enhances RNMT expression and methyltransferase activity [115]. RAM expression is also promoted by eIF4E [122]. eIF4E directly binds RAM mRNA to promote nuclear export of RAM mRNA; however, unlike the eIF4E interaction with RNMT mRNA, this does not increase translation of RAM in the cell system in which it was investigated. RAM mRNA lacks certain motifs in the 5′ UTR that are present in RNMT mRNA, such as the RPS3-, RMX- and YBX1-binding elements, which may explain why eIF4E does not target RAM transcripts for translation.

RAM is regulated by phosphorylation and ubiquitination [116]. RAM is phosphorylated at Ser36 by ERK1/2 kinases during embryonic stem cell differentiation [116]. RAM can only be phosphorylated by ERK1/2 as a monomer; RAM in complex with RNMT cannot be phosphorylated on Ser36. Ser36 phosphorylation targets RAM for ubiquitination and proteasomal degradation, including during differentiation of embryonic stem cells [116]. Since ERK1/2 activity is suppressed by pluripotency factors, RAM (and to some extent RNMT) expression is indirectly regulated by OCT4 and SOX2.

## Other mechanisms regulating RNMT activity and function

### Transcription factors

Regulation of RNA cap formation was first observed in response to the transcription factors N-Myc and c-Myc [118,119]. Myc mutants defective for DNA binding and gene-specific transcription retain the ability to selectively increase the translation of Myc target genes such as components of TFIIH and P-TEFb [118,120]. Myc DNA-binding mutants also partially rescue proliferation and morphology defects in Myc-null cells. The N-terminal domain of Myc recognises transcription start sites independently of recruitment to E-box sequences in DNA, the canonical Myc-binding sites. The mechanism whereby Myc DNA-binding mutants promote RNA cap formation at these transcripts is unclear, but the Myc transactivation domain may activate/increase recruitment of transcription regulators with which it interacts, including TFIIH, thus increasing RNA cap formation at specific gene loci.

The transcription factors Myc and E2F1 globally up-regulate RNA cap formation (Figure 6B) [118–121,130]. Human RNA pol II CTD is comprised of 52 tandem heptapeptide repeats that are differentially modified throughout the stages of transcription, co-ordinating recruitment of RNA processing enzymes [131–133]. Phosphorylation of serine-5 (Ser5) and serine-2 (Ser2) of the CTD is associated with transcription initiation and elongation, respectively. Transcription factors Myc and E2F1 promote RNA pol II CTD phosphorylation at these sites by promoting recruitment of the CTD kinases TFIIH and P-TEFb [118,134]. Myc-dependent RNA pol II CTD phosphorylation contributes to transcription and recruitment of RNA processing enzymes including RNGTT [118,120,130]. RNGTT recruitment and activity is stimulated by the phosphorylated RNA pol II CTD, enhancing inverted guanosine addition to the nascent transcript [24,25,124,125]. Increased guanosine cap formation is required prior to RNA cap methylation by RNMT and other cap methyltransferases. Hence, Myc and E2F1 can indirectly promote cap methylation via RNA pol II CTD phosphorylation and RNGTT recruitment and activation [120,130].

Myc also promotes RNMT function by up-regulating *S*-adenosyl homocysteine hydrolase (SAHH) [121,135]. Transfer of the methyl group from SAM to the guanosine cap generates the by-product SAH. SAH accumulation inhibits cap methyltransferase reactions by competing with SAM for the active site, preventing guanosine cap binding and methylation [107,136]. SAHH hydrolyses SAH to adenosine and homocysteine, derepressing RNMT and permitting SAM binding [137]. Up-regulation of SAHH is also likely to improve the function of other methyltransferases. Myc-induced up-regulation of SAHH is required for Myc-induced cap methylation. Other regulators of SAHH are also likely to promote cap methylation. Of note, SAHH is up-regulated during T cell activation [135].

### eIF4E

The cap-binding protein, eIF4E, influences RNMT expression by several mechanisms [122]. In addition to the role of eIF4E as a translation initiation factor, eIF4E facilitates the nuclear export of specific transcripts via the eIF4E sensitivity element (4ESE) in the 3′ UTR [138,139]. Independently of nuclear export, eIF4E also selectively increases the translation efficiency of transcripts with highly structured 5′ UTRs [140]. eIF4E facilitates both nuclear export and translation of *RNMT* mRNA, promoting RNMT expression and RNA cap formation [122].

Independently of eIF4E-mediated up-regulation of RNMT expression, eIF4E also directly interacts with RNMT protein [141]. eIF4E and RAM-binding sites overlap on the RNMT methyltransferase domain. Unlike RAM, eIF4E has not been observed to promote methyltransferase activity. eIF4E may compete with RAM in cells to regulate RNMT methyltransferase activity, but further studies will be required to test this hypothesis. The RNMT-eIF4E interaction is also independent of cap binding. As eIF4E has a higher affinity for fully methylated caps compared with RNMT, the RNMT-eIF4E nuclear interaction may facilitate direct transfer of newly capped transcripts for targeted nuclear export by eIF4E. Whether there are additional cytoplasmic functions of the RNMT-eIF4E complex remains unexplored.

### RNMT Thr77 phosphorylation

Post-translational modification of the RNMT N-terminus is a mechanism by which RNA cap methylation is regulated [114]. The RNMT N-terminal domain is important for chromatin recruitment but also inhibits catalysis [111]. Phosphorylation of threonine-77 (Thr77) within the RNMT N-terminus both directly and indirectly increases cap methyltransferase activity [112,114]. RNA cap methylation is up-regulated by RNMT Thr77 phosphorylation *in vitro* and in cells. Since the structure of the RNMT N-terminus has not been solved, there is limited molecular insight into how methyltransferase activity is repressed by the N-terminal domain and promoted by Thr77 phosphorylation. AlphaFold modelling predicts that Thr77 lies in an unstructured region distal to the potential N-terminal α-helix (Figure 4C) [109,110]. Thr77 phosphorylation also represses the RNMT-RAM interaction with importin-α. Importin-α binding inhibits RNMT methyltransferase activity (discussed later).

Thr77 phosphorylation is cell–cycle dependent, occurring during late S and G2/M phases [112,114]. Thr77 is phosphorylated by the cyclin-dependent kinase (CDK) CDK1-Cyclin B1, the predominant cyclin-dependent kinase active during this period of the cell cycle [142]. Thr77 phosphorylation during late S and G2/M provides increased RNA cap methyltransferase activity for the following G1 phase of the cell cycle when most transcription occurs [143]. Other kinases may also phosphorylate RNMT Thr77, including other CDKs. Of note, inhibition of the RNA pol II CTD kinases does not result in a reduction in RNMT Thr77 phosphorylation, suggesting that these kinases do not phosphorylate RNMT despite their proximity during transcription [112]. Other RNMT post-translational modifications have been identified but not characterised [114].

### Importin-α

RNMT interacts with several importin proteins, including importin-α, as discussed [112,113]. RNMT is inhibited by its interaction with importin-α and potentially other importins [112]. The RNMT-importin-α interaction is dependent on the basic RNMT N-terminal residues 96–143 [112,113]. The mechanism by which importin-α inhibits RNMT is unclear but is direct since it is observed with recombinant proteins. This interaction is blocked by Thr77 phosphorylation which may alter the conformation of RNMT to inhibit binding without directly interacting with importin-α residues. Since Thr77 phosphorylation occurs during the G2/M phase, the RNMT-importin-α interaction is inhibited at this time [112]. RNMT is predominantly nuclear and methylates caps on nascent RNA; however, the RNMT-importin-α interaction may inhibit RNMT function in the cytoplasm following nuclear envelope breakdown during mitosis.

## Roles of RNMT in cellular function

### Proliferation

RNMT is required for cell proliferation. Inhibition of RNMT expression or function reduces cell proliferation rates and induces apoptosis across many cell lineages, including HeLa cells, breast cancer cell lines and primary T cells [79,102,112,114,115]. The RNMT co-activator RAM promotes cell survival and proliferation [28,29].

The Myc family of transcription factors is a major conduit for cell proliferation signals. Myc proteins are up-regulated in response to growth factors to regulate the expression of target genes that collectively promote proliferation [144–146]. Myc up-regulates RNA pol II phosphorylation to promote RNA cap formation [118,119,135]. As discussed, DNA binding-deficient Myc mutants partially rescue cell proliferation defects in Myc-null cells, as the Myc N-terminus can be recruited to transcription start sites and promote cap methylation for Myc-target gene expression. Myc also up-regulates the enzyme SAHH to neutralise the inhibitory by-product of methylation reactions, SAH [135]. Inhibition of SAHH expression leads to reduced ^m7^G mRNA abundance and proliferation defects. The relationship between RNMT and Myc depends on the cell type. Up-regulation of RNMT expression during activation of CD4 and CD8 T cells is Myc-dependent [79]. Conversely, RNMT-RAM is required for Myc expression in HeLa cells [123]. However, in breast cancer cell lines and T cells, Myc expression does not alter upon RNMT knockdown [102].

RNMT enzymatic activity is increased in a cell cycle-dependent manner to promote cell proliferation [112,114]. As discussed, RNMT is phosphorylated by CDK1-Cyclin B1 at Thr77 enhancing cap methyltransferase activity. RNMT Thr77 is phosphorylated during late S and G2/M phase of the cell cycle, increasing RNMT activity to coincide with the rapid increase in transcription that occurs during G1 phase of the cell cycle. Mutating RNMT Thr77 to alanine prevents phosphorylation and inhibits proliferation in HeLa cells. RNMT Thr77 phosphorylation has a broad effect on gene expression in this context, with changes in levels of proteins involved in many cellular functions. Gene-specific transcript level changes were not detected when Thr77 phosphorylation was blocked in HeLa cells [112], indicating that RNMT phosphorylation is either having a subtle impact on all mRNAs or predominantly promotes RNA processing or protein synthesis.

### RNA translation

When cell proliferation increases, this requires enhanced translation to produce sufficient protein for increased cell mass. While cap guanosine-7 methylation directly affects the translation of mRNAs, RNMT also promotes the synthesis of ribosomal RNA (rRNA), ribosomal proteins (RPs) and other ribosome biogenesis factors to promote cellular proliferation [79,115,123]. RNMT-RAM is directly recruited to the H4 and H13 transcribed sites in ribosomal DNA (rDNA), as well as the RNA pol I transcription of intergenic spacer regions (IGS) [123]. The exact function of RNMT at rDNA loci and how it promotes rRNA synthesis at these sites remains undetermined. As discussed earlier, in certain cell types, RNMT regulates c-Myc expression in HeLa cells [123]. Myc regulates ribosome biogenesis and protein synthesis via a number of mechanisms [147–150]. RNMT thus promotes ribosome biogenesis in part by up-regulating c-Myc expression [123]. Nucleolar RNA pol II also supports rRNA biogenesis in human cells by preventing nucleolar condensate destabilisation related to RNA pol I transcription of intergenic spacer regions (IGS) [151,152].

### T cells: RNA translation and proliferation

RNMT-RAM up-regulates ribosome biogenesis in T cells to meet demands for increased protein synthesis upon T cell activation and population expansion [79,115]. T cells are important for adaptive immunity and comprise two major subtypes: cytotoxic CD8 T cells that kill infected cells and CD4 helper T cells that support co-ordination of the immune response via the secretion of cytokines and other factors. Naive T cells have low levels of gene expression and metabolism. When a T cell receptor is presented with a cognate antigen and co-receptor stimulation, transcription and translation are up-regulated, leading to rapid proliferation and differentiation into effector T cell populations, in a process called activation. RNMT and RAM are up-regulated during T cell activation, and this is required to increase mRNA levels [79,115]. RNMT-RAM protein up-regulation during T cell activation is Myc-dependent [79]. The transcripts encoding ribosomal proteins and ribosome biogenesis factors, TOP RNAs (described above), are particularly dependent on RNMT-RAM. RNMT-RAM up-regulation during T cell activation increases mRNA production and the ribosome production for mRNA translation. In activated T cells, RNMT-RAM do not up-regulate Myc expression, which is already highly expressed following T cell activation [79,153,154].

When either RNMT or RAM is knocked out in T cells during thymic development, fully capped mRNAs are still detected for the first few days after T cell activation. RNMT and RAM are stable proteins and it is likely that trace RNMT-RAM is present for initial cell divisions after T cells are activated, permitting low level cap methylation until apoptosis occurs. In *Rnmt* knock-out T cells, transcripts initiating with C (including TOP mRNAs) retain the ^m7^G cap, whereas other transcripts have a mixture of ^m7^G cap and G caps. The enrichment of ^m7^G caps on TOP mRNAs indicates that RNMT has a special relationship with C-capped RNA. The non-methylated cap on these C-cap transcripts is likely to be the most unstable and targeted for degradation, consistent with loss of LARP1 binding and stabilisation. TOP mRNAs are particularly sensitive to the disruption of post-transcriptional cytoplasmic cap methylation, indicating that TOP mRNAs undergo cycles of decapping and recapping (see later) within the cytoplasm [155]. RNMT knockout thus destabilises TOP mRNAs both by inhibiting cap homeostasis (see later), and by preventing LARP1-mediated protection from degradation.

### Embryonic stem cell differentiation

Differentiation of Embryonic Stem Cells (ESCs) involves co-ordinated regulation in gene expression to direct changes in cell fate. ESCs have high levels of RNMT and RAM, and RNMT is regulated via RAM during differentiation of ESCs [116,128]. RAM-sensitive transcripts include genes associated with pluripotency and development, including Oct4, Sox2 and Klf2. ESCs require RAM to maintain the high expression of these pluripotency factors. During neuronal differentiation, ERK1/2 expression and phosphorylation increase, co-ordinating many cellular events required for differentiation via phosphorylation of substrates [156,157]. ERK1/2 phosphorylates RAM on Ser36, targeting it for ubiquitination and degradation, and reinforcing repression of pluripotency associated proteins. Repression of RAM is important for differentiation; experimental maintenance of high RAM levels blocks differentiation. Hence, RNMT-RAM contributes to the robustness of the pluripotency network, where high levels of RAM maintain pluripotency factor expression above a threshold [116]. Repression of pluripotency factors breaks this cycle of RNMT-RAM and pluripotency gene expression and contributes to robust differentiation.

### Post-transcriptional capping

Formation of the ^m7^G cap occurs predominantly co-transcriptionally to protect RNA pol II transcripts during synthesis and promote co-transcriptional processing and nuclear export. Post-transcriptional capping or ‘recapping’ is observed in the cytoplasm of human cells and may occur at the original 5′ site or at downstream sites generated by endonucleolytic cleavage to produce stable 5′ truncated transcripts [158,159]. Post-transcriptional capping contributes to cap homeostasis [155,160], and the cellular response to stress [161,162], with limited evidence supporting wholescale changes in proteomic complexity [163].

Post-transcriptional capping was first described for β-globin, where stable 5′ truncated transcripts were exclusively detected in the cytoplasm [164,165]. These transcripts possess a cap-like structure at their 5′ ends that is recognised by a trimethyl cap antibody, binds to eIF4E and is susceptible to DCP2 digestion [161,166]. Later work identified similarly 5′-modified sites downstream of transcription start sites throughout the transcriptome, even occurring in the 3′ UTR [155,167–169]. As downstream 5′-modified and cytoplasmically capped sites do not always correlate, there may be additional cap-like structures downstream of the transcription start site [159,169]. Although mRNA has been the major focus of the recapping field, other RNA pol II transcripts such as lncRNAs also contain downstream 5′-modified sites [170]. Inhibition of cytoplasmic capping enzymes facilitated the detection of transcripts undergoing cytoplasmic recapping at the original 5′ site and at downstream sites [155,160].

Post-transcriptional capping is catalysed by RNGTT and RNMT-RAM [127,161]. Decapping generates RNA with a 5′ monophosphate end [171] which needs to be converted to a 5′ diphosphate for recapping by RNGTT [161,170]. The 5′ monophosphate kinase involved in cytoplasmic capping is unknown. The cytoplasmic capping complex also includes the adapter protein NCK1 as a scaffold [170]. NCK1 contains three SH3 domains that directly bind to both the kinase and RNGTT. RNGTT binds to the SH3 domain via its proline-rich C-terminus. Although the kinase and RNGTT do not directly interact, RNMT-RAM is recruited to the cytoplasmic capping complex via direct interactions with RNGTT [127]. The cytoplasmic capping complex may be very low abundance in cells as it falls under the threshold for detection by mass spectrometry [172].

Post-transcriptional capping is required for cap homeostasis, the cyclical process of decapping and recapping to maintain transcript stability and capacity for translation [160]. Transcripts sensitive to changes in cytoplasmic capping are involved in pathways including nucleotide binding, protein localisation, RNA metabolic proteins, the mitotic cell cycle and translation regulation via the TOP mRNAs [155,160]. Multiple mechanisms have been put forward to explain the selectivity of cytoplasmic recapping, including AU-rich sequences in the 3′ UTR [160], alternative splicing [173], and RNA-binding protein-mediated recruitment [172]. The contribution of RNMT-RAM to cap homeostasis was investigated using a cytoplasmically-restricted, catalytic-dead construct lacking the N-terminal regulatory domain [172]. Expression of this RNMT catalytic mutant led to a decrease in a subset of transcripts enriched for the complete cap structure, such as TOP mRNAs; hence, ^m7^G cap methylation is necessary for transcript stability in cap homeostasis. ^m7^G cap methylation is also required for the translation of cytoplasmic capping targets [174].

### Cancer

Cancer encompasses many diseases in which cells undergo malignant transformation to proliferate in a dysregulated manner. Pan-cancer analysis shows that RNMT expression is associated with prognosis in certain cancer types [175,176]. RNMT expression is associated with expression of certain oncogenes. RNMT is up-regulated in high-eIF4E acute myeloid leukaemia patients, elevating capping for genes including Myc, β-catenin and MDM2 [122]. Elevated expression of RNMT, c-Myc and B7-B7 homologue 6 (B7-H6) in glioma stem-like cells and tissue samples is associated with cell proliferation [177].

RNMT-RAM is associated with cell transformation. Regulation of RNA cap methylation as a mechanism of gene regulation was first reported in response to the Myc transcription factors [118]. RNMT overexpression induces transformation of immortalised human mammary epithelial cells, permitting anchorage-independent proliferation, presumably due to dysregulation of key target genes [101]. The ability of deregulated Myc proteins (or Ras) to promote cell transformation is also increased by elevated expression of RNMT [101].

RNMT targeting in a panel of breast cancer cell lines induces apoptosis [102]. Breast cancer cell lines with oncogenic PI3KCA mutations are most sensitive to RNMT inhibition. Furthermore, the expression of oncogenic PIK3CA mutations sensitises cells to RNMT inhibition. Expression of oncogenic PIK3CA mutants does not alter RNMT expression and activity; therefore, it is possible that the contribution of RNMT to other cancer types may have been overlooked in the absence of RNMT expression deregulation. Given the critical role of RNMT in gene expression, cell proliferation and cell transformation, RNMT is a promising candidate for cancer therapeutics. Tumours with oncogenic mutations in c-Myc and PIK3CA may be most sensitive to RNMT targeting.

RNMT contributes to immune phenotypes in cancer [175,176]. Tumours exhibit multiple strategies for evasion of anti-tumour immunity responses such as by preventing immune cell infiltration [178,179]. RNMT expression is associated with tumour infiltration of B cells, CD4 and CD8 T cells, dendritic cells, macrophages and neutrophils [175].

## Discussion

**Figure 7:**
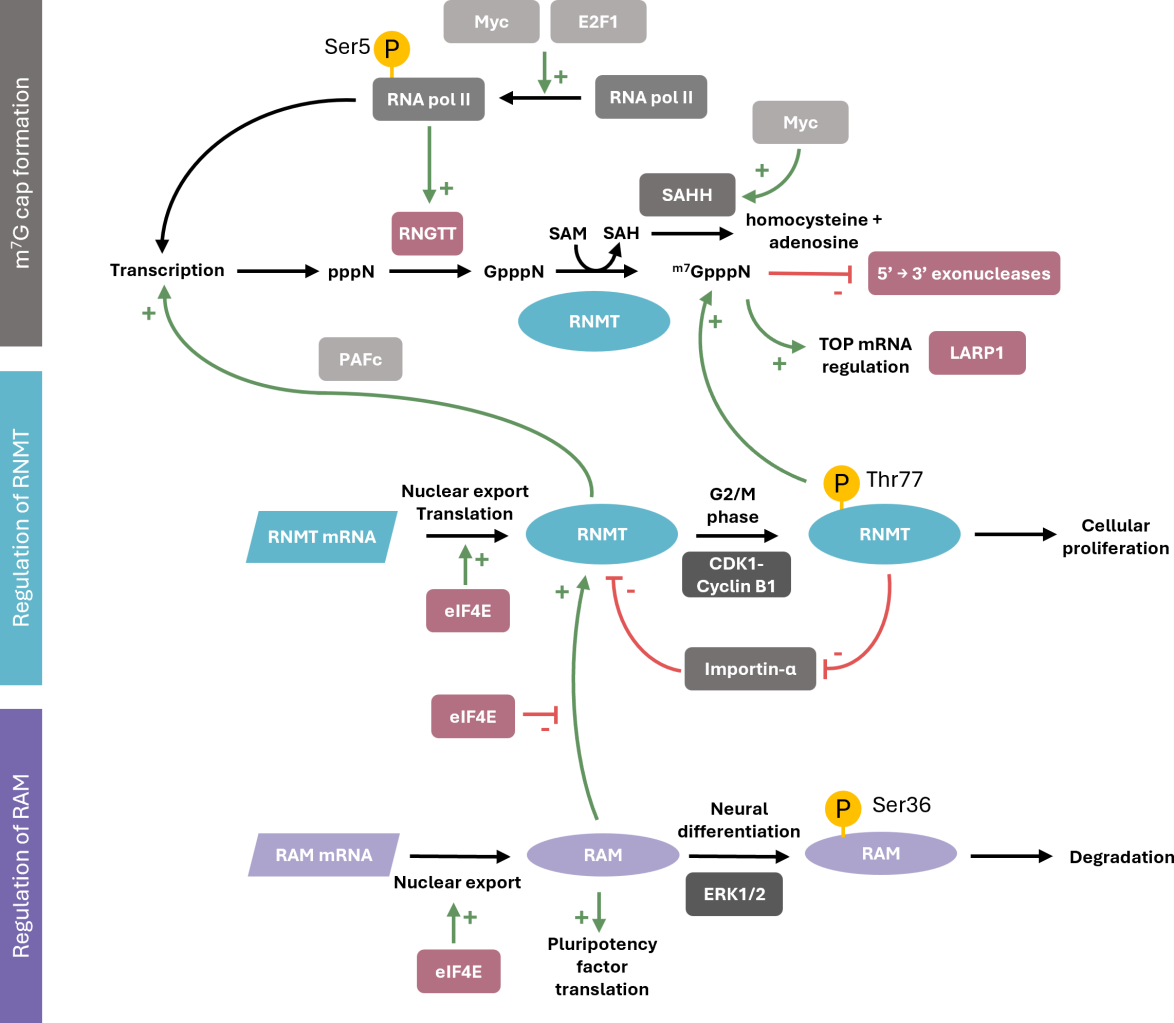
Summary of the regulation of RNMT and its roles in cellular function. RNMT regulation occurs initially during ^m7^G cap formation, whereby regulation in the amount of RNMT substrates (G cap and SAM) by Myc, E2F1, and RNA pol II alter RNMT enzymatic activity. RNMT is also directly regulated by both post-translational modification and context-dependent interaction partners with consequences on ^m7^G cap formation and cellular phenotypes such as proliferation. Another important mechanism for RNMT regulation is via its co-activator RAM, which is similarly regulated by post-translational modifications and other interaction partners to hinder its interaction with RNMT, thus reducing RNMT methyltransferase activity and stability. (CDK1, cyclin dependent kinase 1; E2F1, E2F transcription factor 1; eIF4E, eukaryotic translation initiation factor 4E; ERK1/2, extracellularly regulated kinases 1/2; LARP1, La-related protein 1; Myc, Myc proto-oncogene, bHLH transcription factor; PAFc, polymerase-associated factor 1 complex; RAM, RNMT activating miniprotein; RNA pol II, RNA polymerase II; RNMT, guanine-7 methyltransferase; RNGTT, RNA guanylyltransferase and 5’ phosphatase; SAH, S-adenosyl homocysteine; SAHH, *S*-adenosyl homocysteine hydrolase; SAM, *S*-adenosyl methionine; TFIIH, transcription factor II H)

In summary, the RNA cap methyltransferase, RNMT, is an integrator of cellular signalling information, receiving information from pathways which affect RNMT or co-factor RAM expression and post-translational modification (Figure 7). Alterations in RNMT function drive changes in transcription, RNA stability, splicing, nuclear export and protein synthesis. The response to regulation of RNMT expression or configuration is gene-specific, often directing changes in cell proliferation, differentiation or function. How regulation of RNMT drives gene-specific changes in expression can be partially explained in terms of the rate of transcription and interaction with cap-binding proteins. As with many gene-specific mechanisms involving RNA, a combination of factors influences RNA abundance and usage. In many cell types, RNMT promotes cell proliferation. As a result, RNMT is investigated as a potential therapeutic target in cancer or immune disorders.

## Abbreviations

CAPAM: cap adenosine N6-methyltransferase
CBC: cap-binding complex
RAM: RNMT-activating miniprotein
RNGTT: RNA guanylyl and 5′-phosphatase
RNMT: RNA N7-methyltransferase
